# De-escalation of regional nodal irradiation fields in pT1-2N1 breast cancer patients after breast conserving surgery: retrospective real-world clinical experience

**DOI:** 10.3389/fonc.2025.1484190

**Published:** 2025-03-21

**Authors:** Ji Hyun Hong, Jin-Ho Song, Kyu-Hye Choi, Shin Woo Kim, Woo-Chan Park, Jieun Lee, Ahwon Lee, Jun Kang, Byung-Ock Choi

**Affiliations:** ^1^ Department of Radiation Oncology, Seoul St. Mary’s Hospital, College of Medicine, The Catholic University of Korea, Seoul, Republic of Korea; ^2^ Division of Breast Surgery, Department of Surgery, Seoul St. Mary's Hospital, College of Medicine, The Catholic University of Korea, Seoul, Republic of Korea; ^3^ Division of Medical Oncology, Department of Internal Medicine, Seoul St. Mary's Hospital, College of Medicine, The Catholic University of Korea, Seoul, Republic of Korea; ^4^ Department of Hospital Pathology, Seoul St. Mary's Hospital, College of Medicine, The Catholic University of Korea, Seoul, Republic of Korea

**Keywords:** breast cancer, regional nodal irradiation, whole breast irradiation, breast-conserving surgery, disease-free survival, overall survival, local-regional recurrence-free survival

## Abstract

**Purpose:**

Regional nodal irradiation (RNI) in pN1 patients with one to three positive axillary lymph node breast cancers remains controversial. This study aimed to evaluate the impact of RNI in patients with pT1-2N1 breast cancer who underwent radiotherapy after breast-conserving surgery (BCS), focusing on risk stratification and defining the extent of RNI as axillary lymph node levels I and II.

**Methods:**

Female patients with pT1-2N1 breast cancer after BCS with axillary lymph node dissection or sentinel lymph node biopsy who were treated with radiotherapy between 2009 and 2021 were identified. Radiotherapy included either whole-breast irradiation (WBI) alone or WBI with RNI to axillary levels I and II. Patients were categorized into three risk groups based on pathological T stage, number of positive lymph nodes, and immunohistochemical classification.

**Results:**

A total of 464 patients were analyzed, with a median follow-up of 68.5 months. A total of 212 (45.7%) patients received WBI alone, and 252 (54.3%) received WBI with RNI. Overall, RNI did not significantly improve disease-free survival (DFS) (p = 0.317), locoregional recurrence-free survival (LRRFS) (p = 0.321), distant metastasis-free survival (DMFS) (p = 0.452), or overall survival (OS) (p = 0.721). However, RNI demonstrated a significant benefit in terms of LRRFS (p = 0.014) in the high-risk group. Case–control matched analysis showed robust benefits in DFS (p = 0.020), LRRFS (p = 0.030), and marginal improvement in DMFS (p = 0.066) in the high-risk group. The toxicities were comparable between WBI alone and WBI with RNI.

**Conclusions:**

RNI omission may be considered in low-risk patients with pT1 and one positive lymph node. High-risk patients with pT2, two to three lymph nodes, or triple-negative breast cancer may benefit from RNI. De-escalation of the RNI extent might be considered for non-inferior survival outcomes with comparable toxicities.

## Introduction

In breast cancer patients with one to three positive axillary lymph nodes, several prospective randomized trials and meta-analyses have reported loco-regional outcomes and survival benefits favoring adjuvant regional nodal irradiation (RNI) in addition to whole-breast irradiation (WBI) after breast-conserving surgery (BCS) or post-mastectomy radiation (PMRT) after mastectomy (1–3). Advances in treatment and diagnostic techniques, including chemotherapy, hormonal treatment, surgery, and radiation therapy, have improved recurrence rates and survival outcomes. For instance, anthracycline plus taxane-based chemotherapy has shown better disease-free survival (DFS) in node-positive breast cancer than anthracycline alone (4), and the addition of taxanes, endocrine therapy, and anti-human epidermal growth factor receptor 2 (HER2) treatment reduces locoregional recurrence (5–8).

However, major clinical trials supporting postoperative radiotherapy have not accounted for these advances (9). The uncertainty regarding the impact of systemic therapy and radiotherapy has fueled the idea of de-escalating certain aspects of radiotherapy. Due to these improvements, the perceived benefits of RNI may no longer be as significant as before. Several studies (10–13) have evaluated or are still evaluating the benefit of RNI in patients with pN1 breast cancer. Additionally, both axillary lymph node dissection (ALND) and RNI can increase toxicity, leading to lymphedema and pneumonitis (6, 14, 15), which negatively affects the quality of life. Furthermore, intensity-modulated radiation therapy (IMRT), a new modality of radiotherapy, has become increasingly popular (16, 17). Axillary coverage by whole-breast irradiation shows notable differences between IMRT and standard 3-dimensional tangential plans (18). Given the ongoing uncertainty regarding the effects of systemic therapy and the increased toxicity associated with broader radiation fields, de-escalation of postoperative radiotherapy may be considered for selected patients.

Although several studies have investigated the use of RNI in pN1 patients with one to three positive axillary lymph nodes pathologically (12, 13, 19), evidence from randomized trials is still lacking. An ongoing prospective multicenter randomized phase 3 trial in patients with pN1 breast cancer (PORT-N1; Korean Radiation Oncology Group 22-05; NCT05440149) (11) aimed to evaluate the feasibility of postoperative radiotherapy (RT) de-escalation. In the PORT-N1 trial, patients with pN1 breast cancer who underwent BCS and mastectomy were included and randomly assigned to either the WBI or RNI/PMRT group or the WBI alone/no PMRT group. While awaiting results from the PORT-N1 trial and serving as a bridging retrospective study for prospective studies omitting RNI, we aimed to highlight the difference between radiation fields designed with or without the intention of delivering the prescribed radiation dose, investigate whether there is a significant risk of outfield recurrence in regional lymph nodes, such as supraclavicular lymph nodes (SCN), and evaluate our retrospective data to identify which group of pN1 patients should be considered for omitting RNI, and whether defining the extent of de-escalated RNI affects survival and loco-regional outcomes.

## Materials and methods

Female patients with pT1-2N1 breast cancer treated with RT after BCS at a single institution between 2009 and 2021 were included in the analysis. Eligible patients underwent BCS with ALND or sentinel lymph node biopsy (SLNB). All patients were pathologically confirmed to have one to three positive lymph nodes and treated with postoperative RT. RT included either RNI to axillary levels I and II in addition to WBI or WBI alone. Patients were excluded if they had coexisting cancers other than thyroid cancer, a history of radiotherapy, recurrent ipsilateral breast cancer, or had undergone neoadjuvant chemotherapy, as neoadjuvant chemotherapy can downstage the nodal stage and the optimal axillary treatment following neoadjuvant chemotherapy remains unclear (20). Additionally, patients who received radiotherapy to the SCN and axillary level 3 were excluded, as pN1 disease primarily involves lower axillary metastasis and covering level 2 might be sufficient. Therefore, this study selectively targeted axillary levels I and II, focusing on comparing WBI alone with WBI with axillary levels I and II.

Radiotherapy was administered using a photon beam with an energy range of 4 MV–10 MV. Three-dimensional conformal radiotherapy (3D-CRT) and IMRT were performed. Either electron or photon beams were used for the tumor bed boost irradiation. All patients underwent computed tomography (CT)-based simulations. Patients were treated with either hypofractionated regimens, delivering 40 Gy–50 Gy with a dose per fraction of 2.67 Gy, or standard fractionation schemes, delivering 59.4 Gy–64.8 Gy with a dose per fraction of 1.80 Gy.

As genomic tests such as Oncotype DX, MammaPrint, and EndoPredict were not routinely performed during the study period, we stratified patients into three risk groups based on pathologic T stage, number of positive nodes, and immunohistochemical classification, as in previous studies (10, 21, 22). The low-risk group included patients with T1 stage tumors and one positive lymph node. The intermediate-risk group comprised tumors with a T1 stage and two to three positive lymph nodes, T2 stage tumors and one positive lymph node, or T1 stage tumors with estrogen receptor (ER) positive and HER2 positive tumor. The high-risk group included tumors with T2 stage and two to three positive lymph nodes or tumors proven to be immunohistochemically triple-negative breast cancer (TNBC).

The primary outcome was disease-free survival (DFS); secondary outcomes were overall survival (OS), loco-regional recurrence-free survival (LRRFS), and distant metastasis-free survival (DMFS). The time of origin of all survival outcomes was defined as the date of BCS. DFS and OS were evaluated from the date of BCS to the date of recurrence of breast cancer or breast cancer-specific death. LRRFS was described by the time from BCS to breast cancer recurrence in the ipsilateral breast, axilla, supra/infraclavicular lymph nodes, or internal mammary lymph nodes. DMFS was defined as the time from BCS to the radiological and/or pathological evidence of distant breast cancer.

Patient charts were reviewed, and the treatment and patient characteristics of those who received WBI with RNI to axillary levels I and II were compared to those who received WBI alone using a chi-squared or t-test based on variable characteristics. Survival analyses were performed using the Kaplan–Meier method and compared using the log-rank test. Prognostic factors associated with survival outcomes were analyzed using a multivariate Cox regression model. The variables selected in the multivariate models were determined using covariates (p <0.1) in univariate analysis and previous studies (21, 22). Logistic regression was used for the toxicity analysis. Statistical significance was set at p <0.05.

We also conducted case–control matching analysis. The control group comprised patients who underwent WBI alone. All statistical analyses were performed using STATA/SE (version 17.0; StataCorp, LLC). The Institutional Review Board of Seoul St. Mary’s Hospital (Number: KC23RISI0923) reviewed and approved this study. The requirement for patient consent for inclusion was waived because of the retrospective nature of the study.

## Results

Among 4,651 patients who received RT for breast cancer at our institution between May 2009 and December 2021, 782 were pathologically proven to have T1–2 and one to three positive lymph nodes. After excluding 318 patients who received neoadjuvant chemotherapy, underwent mastectomy, had only ALND without excision of the primary tumor, had a lack of medical records, had cancers other than thyroid cancer, were male, had a previous RT history on the breast, had RT fields including SCN, intramammary node, or axilla level III, and had recurrent tumors, a total of 464 patients who met the eligibility criteria were analyzed (**Supplementary Figure S1**). The median follow-up was 68.5 months (range: 9.0–176.2 months) and the median age was 51.5 years (range: 24.4–82.5 years). Radiation treatment was performed on the breast alone in 212 patients (45.7%) and on the breast with axillary lymph node levels I–II in 252 (54.3%). Patient and treatment characteristics are summarized according to the radiation field in **Table 1**.

**Table 1:** Characteristics of patients and treatments.

	WBI, n = 212 (45.7%)		RNI, n = 252 (54.3%)		
	N, median	%, range	N, median	%, range	P-value
Age at RT	50.7	24.35–78.47	52.8	29.31–82.46	0.024
Hypertension	31	14.62	53	21.03	0.074
Diabetes	15	7.08	24	9.52	0.344
Histology					0.023
IDC	192	90.57	209	82.94	
ILC	3	1.42	14	5.56	
others	17	8.02	29	11.51	
T stage					0.146
T1	117	55.19	122	48.41	
T2	95	44.81	130	51.59	
# of tumors					0.126
Single	188	88.68	211	83.73	
Multiple	24	11.32	41	16.27	
Lymphovascular invasion					0.551
Negative	114	53.77	128	51.00	
Positive	98	46.23	123	49.00	
Grade					0.588
1	40	18.87	43	17.13	
2	103	48.58	134	53.39	
3	69	32.55	74	29.48	
Margin status					0.003
Negative	164	77.36	158	62.95	
Close	44	20.75	82	32.67	
Positive	4	1.89	11	4.38	
Lymph node dissection type					<0.001
SLNB	42	19.81	178	70.63	
ALND	170	80.19	74	29.37	
Number of positive macroscopic nodes					0.155
1	153	72.17	167	66.27	
2	42	19.81	51	20.24	
3	17	8.02	34	13.49	
Hormone Receptor (+)	165	77.83	213	84.52	0.065
HER2 (+)	37	17.45	30	11.90	0.086
TNBC	37	17.45	30	11.90	0.090
RT technique					<0.001
3D-CRT	185	87.26	74	29.37	
IMRT	27	12.74	178	70.63	
RT dose (cGy)					<0.001
4000 – 4500	6	2.83	24	9.52	
5005	31	14.62	173	68.65	
5940	162	76.42	50	19.84	
6480	13	6.13	5	1.98	
fraction size (cGy)					<0.001
180	175	82.55	55	21.83	
267	37	17.45	197	78.17	
Adjuvant chemotherapy					0.004
No	40	18.87	77	30.56	
Yes	172	81.13	175	69.44	
Post RT chemotherapy					0.019
No	191	90.09	241	95.63	
Yes	21	9.91	11	4.37	
Anti-HER2 treatment					0.375
No	179	84.43	220	87.30	
Yes	33	15.57	32	12.70	

WBI, whole breast irradiation; RNI, regional nodal irradiation; RT, radiotherapy; IDC, invasive ductal carcinoma; ILC, invasive lobular carcinoma; SLNB, sentinel lymph node biopsy; ALND, axillary lymph node dissection; HER2, human epidermal growth factor receptor-2; TNBC, triple-negative breast cancer; 3D-CRT, 3D conformal radiotherapy; IMRT, intensity modulated radiotherapy.

Patients treated with RNI were a median of 2.1 years older, significantly less likely to have invasive ductal carcinoma (90.6% vs. 82.9%, p = 0.023), had fewer negative margins for the primary tumor from initial surgery (77.4% vs. 63.0%, p = 0.003) although every patient with positive resection margins underwent re-operation, and underwent more SLNB than ALND (19.8% vs. 70.6%, p <0.001). Radiation doses for those in the RNI group were lower with a larger fraction size, which followed hypofractionated schemes (17.5% vs 78.2%, p <0.001), and were performed using more intensity-modulated radiation therapy techniques (12.7% vs. 70.6%, p <0.001). In addition, patients in the RNI group were less frequently treated with adjuvant chemotherapy (81.1% vs. 69.4%, p = 0.004) and post-RT chemotherapy (9.9% vs. 4.4%, p = 0.019).

### Survival outcomes by RNI in entire study population

**Figure 1:**
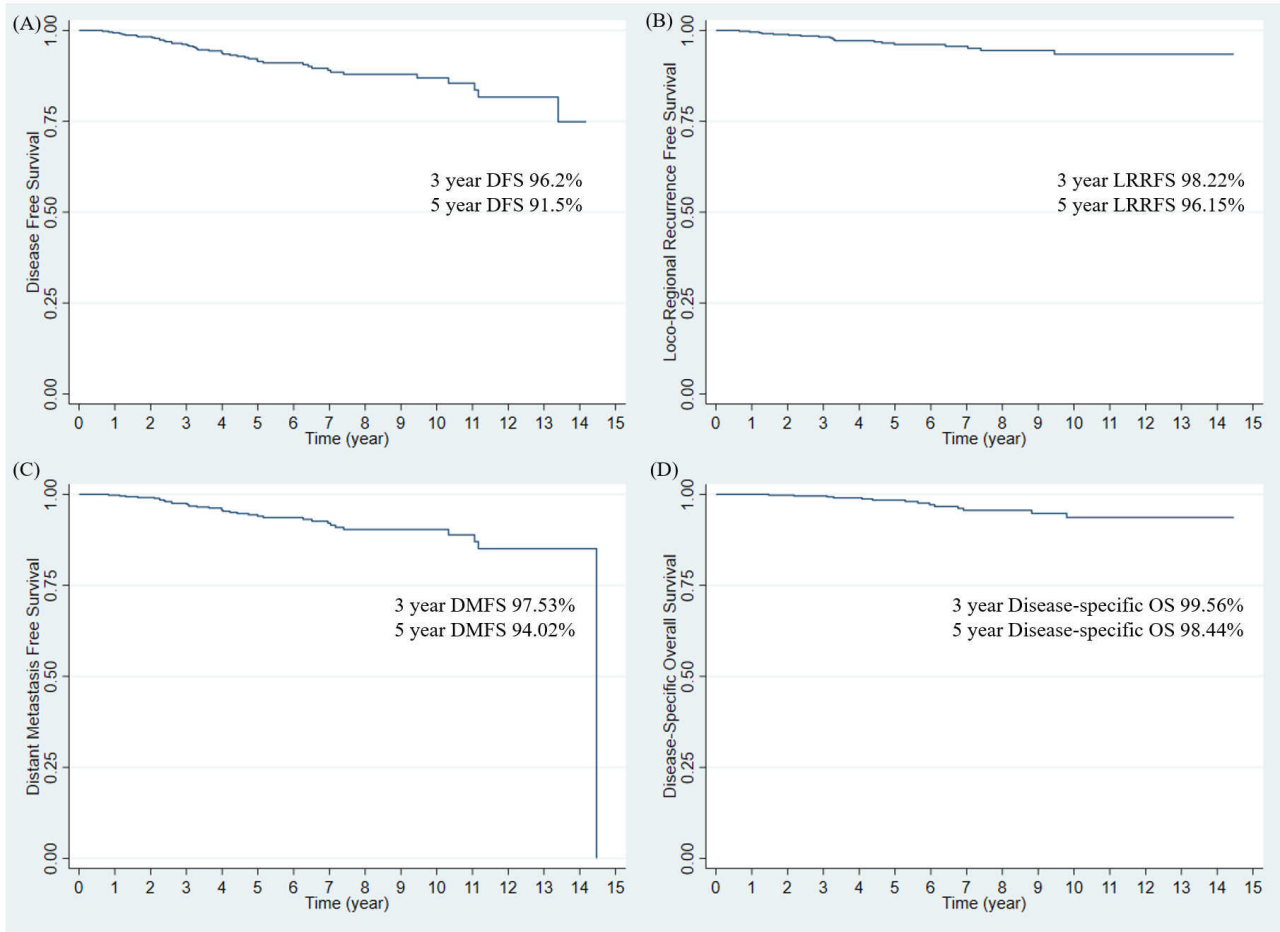
Survival outcomes in entire study population. **(A)** Disease-free survival, **(B)** Locoregional recurrence-free survival, **(C)** Distant metastasis-free survival, and **(D)** Disease-specific Overall Survival. DFS, disease-free survival; LRRFS, locoregional recurrence-free survival; DMFS, distant metastasis-free survival; OS, overall survival.

**Figure 2:**
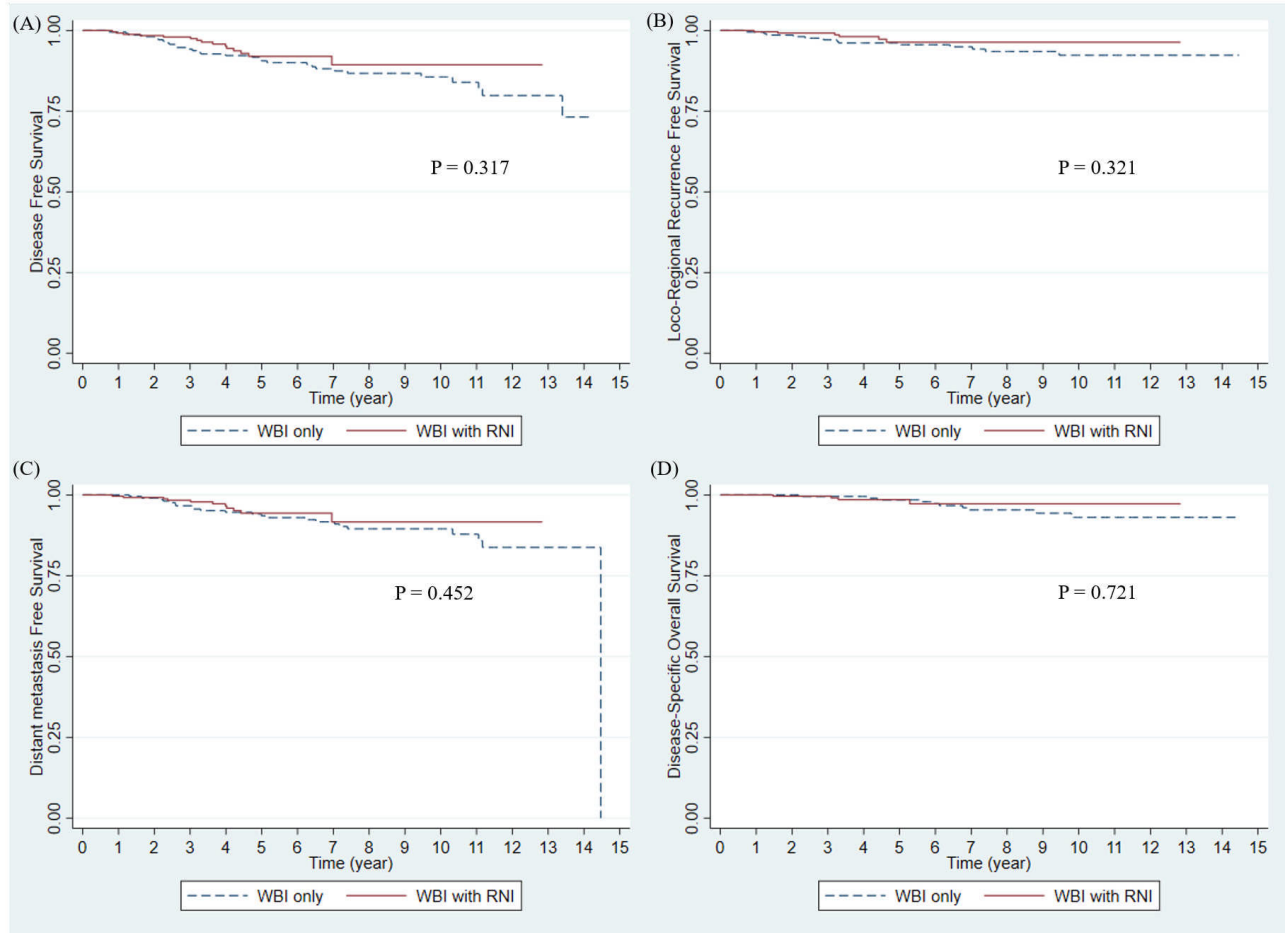
Survival outcomes by RNI in entire study population. **(A)** Disease-free survival, **(B)** Locoregional recurrence-free survival, **(C)** Distant metastasis-free survival, and **(D)** Disease-specific Overall Survival. WBI, whole breast irradiation; RNI, regional nodal irradiation.

Disease progression was observed in 45 (9.7%) patients. The 3- and 5-year DFS rates of the entire study population were 96.2% and 91.5%, respectively (**Figure 1**). Pathologic T stage (T2 vs T1; hazard ratio (HR): 3.37; 95% confidence interval (CI): 1.65–6.86; p = 0.001) was a significant prognostic factor in the multivariate analysis (**Table 2**). The 3-year DFS rates were 94.2% without RNI and 98.0% with RNI, and the 5-year DFS rates were 90.6% without RNI and 92.0% with RNI (**Figure 2**). The surgery type of LN, SLNB or ALND, was not associated with DFS (p = 0.456). DFS between RT with and without RNI was not significantly different (p = 0.317).

**Table 2:** Multivariate analysis of risk factors for survival outcomes.

	DFS				LRRFS				DMFS				Disease-specific OS			
	HR	p-value	95% CI		HR	p-value	95%CI		HR	p-value	95%CI		HR	p-value	95%CI	
**Age at RT**	0.97	0.105	0.94	1.01	0.97	0.162	0.92	1.01	0.97	0.104	0.93	1.01	0.99	0.657	0.93	1.05
**T stage**																
T1	1.00				1.00				1.00				1.00			
T2	3.37	0.001	1.65	6.86	1.24	0.671	0.46	3.34	7.33	<0.001	2.66	20.16	6.68	0.028	1.23	36.22
**Margin status**																
Negative	1.00				1.00				1.00				1.00			
Close	1.85	0.065	0.96	3.55	2.57	0.055	0.98	6.72	1.98	0.083	0.91	4.30	0.68	0.631	0.14	3.29
Positive	2.01	0.370	0.44	9.21	0.00	1	0.00		2.93	0.188	0.59	14.49	28.05	0.002	3.46	227.57
**Hormone Receptor (+)**	1.38	0.758	0.18	10.45	0.28	0.259	0.03	2.59	483,000,000	<0.001	175,000,000	1,330,000,000	0.31	0.340	0.03	3.39
**TNBC**	3.65	0.229	0.44	29.94	2.72	0.381	0.29	25.42	8,340,00,000	.	.	.	0.44	0.514	0.04	5.16
**Post RT chemo**																
No	1.00				1.00				1.00				1.00			
Yes	1.59	0.288	0.68	3.75	2.66	0.112	0.80	8.90	1.61	0.326	0.62	4.14	1.99	0.267	0.59	6.70
**LVSI**																
No	1.00				1.00				1.00				1.00			
Yes	0.78	0.436	0.41	1.46	2.13	0.169	0.72	6.28	0.63	0.210	0.30	1.30	0.35	0.070	0.11	1.09
**Grade**																
1	1.00				1.00				1.00				1.00			
2	0.72	0.457	0.30	1.72	1.37	0.691	0.29	6.58	0.52	0.233	0.18	1.51	1.22	0.878	0.09	16.08
3	0.58	0.321	0.19	1.71	0.33	0.299	0.04	2.70	0.70	0.563	0.21	2.33	11.91	0.064	0.87	163.09
**Number of positive macroscopic nodes**																
1	1.00				1.00				1.00				1.00			
2	0.84	0.651	0.39	1.79	0.63	0.485	0.17	2.29	0.88	0.777	0.37	2.10	3.01	0.114	0.77	11.83
3	0.87	0.770	0.33	2.26	1.08	0.910	0.29	4.02	0.89	0.825	0.30	2.60	4.19	0.036	1.10	15.95

DFS, disease-free survival; LRRFS, locoregional recurrence-free survival; DMFS, distant metastasis-free survival; OS, overall survival; RT, radiotherapy; TNBC, triple-negative breast cancer; LVSI, lymphovascular invasion.

Nineteen patients (4.09%) had showed loco-regional recurrence (LRR). The 3- and 5-year LRRFS rates in the entire study population were 98.22% and 96.15%, respectively (**Figure 1**). In multivariate analysis, the margin status of the primary tumor from the initial surgery (negative vs. close; HR: 2.57; 95% CI: 0.98–6.72; p = 0.055) was a marginally significant prognostic factor (**Table 2**). The 3-year LRRFS rates were 97.13% without RNI and 99.20% with RNI, whereas the 5-year LRRFS rates were 95.58% without RNI and 96.37% with RNI (**Figure 2**). Surgery type of LN either SLNB or ALND, was not associated with LRRFS (p = 0.731). The RT fields (with and without RNI) did not show any significant differences (p = 0.321).

A total of 34 patients (7.33%) showed distant metastasis (DM). The 3- and 5-year DMFS rates in the entire study population were 97.53% and 94.02%, respectively (**Figure 1**). On multivariate analysis, pathologic T stage (T2 vs T1; HR: 7.33; 95% CI: 2.66–20.16; p <0.001) and hormonal receptor (HR: 4.83 × 10^8^; 95% CI: 1.75 × 10^8^–1.33 × 10^9^; p <0.001) were significant prognostic factors (**Table 2**). The 3-year DMFS rates were 96.63% without RNI and 98.35% with RNI, whereas the 5-year DMFS rates were 93.53% without RNI and 94.34% with RNI (**Figure 2**). Surgery type of LN, either SLNB or ALND, was not associated with DMFS (p = 0.143). The RT fields (with and without RNI) did not show any significant differences (p = 0.452).

Seventeen deaths (3.64%) occurred. Among these 17 deaths, 14 were due to disease progression, one was a suicide 1 month after RT, and the other two were from unknown causes. The 3- and 5-year disease-specific OS rates were 99.56% and 98.44%, respectively (**Figure 1**). In multivariate analysis, pathologic T stage (T2 vs T1; HR: 6.68; 95% CI: 1.23–36.22; p = 0.028), margin status of the primary tumor from the initial surgery (negative vs positive; HR: 28.05; 95% CI: 3.46–227.57; p = 0.002), and the number of positive nodes (one vs three; HR: 4.19; 95% CI: 1.10–15.95; p = 0.036) were significant prognostic factors (**Table 2**). The 3-year OS rates were 99.52% without RNI and 99.60% with RNI, whereas the 5-year OS rates were 98.48% without RNI and 98.54% with RNI (**Figure 2**). The surgery type of LN, SLNB or ALND, was not associated with OS (p = 0.171). The RT fields (with and without RNI) did not show any significant differences (p = 0.721).

### Survival outcomes in different risk groups

**Figure 3:**
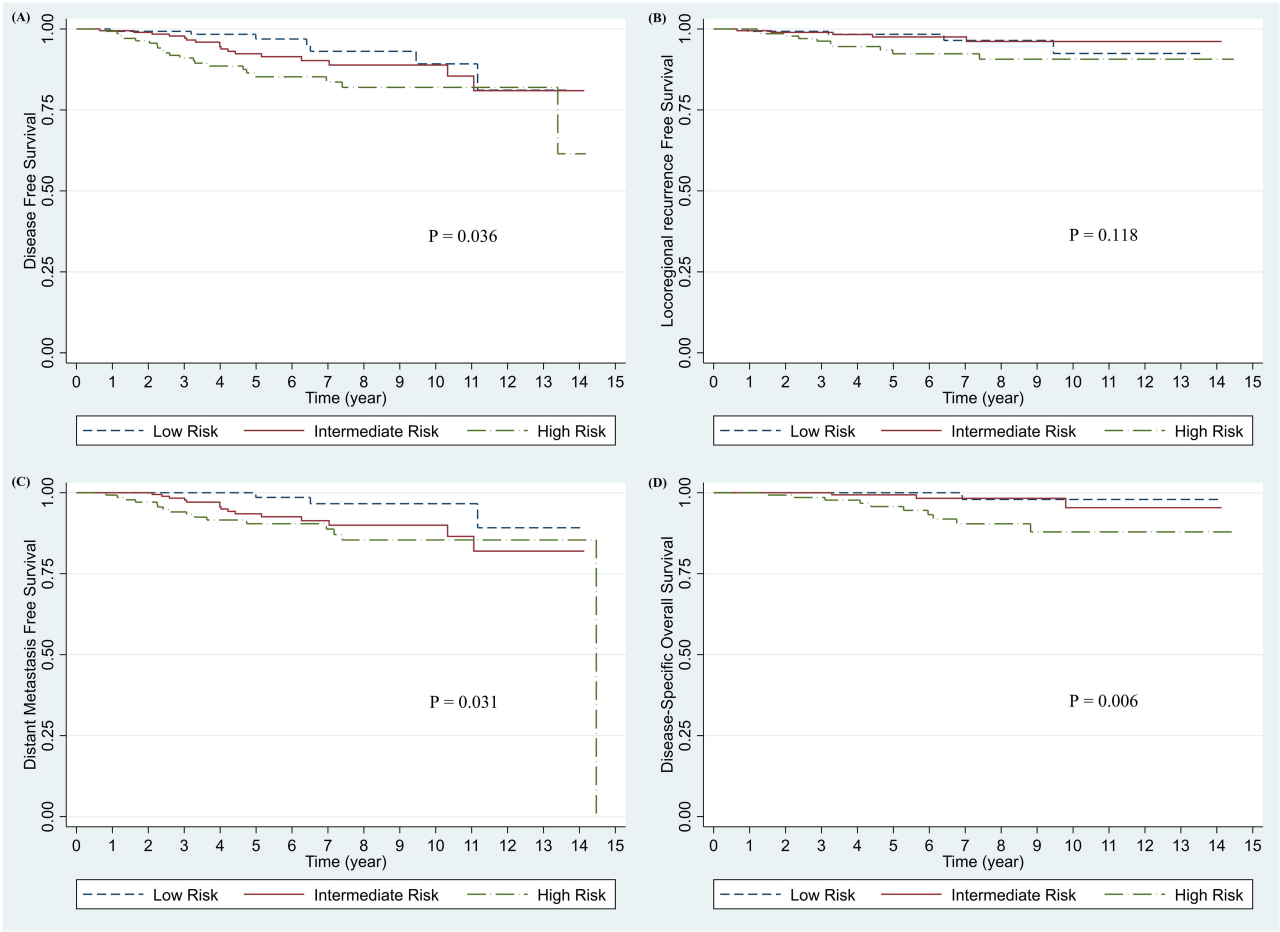
Survival outcome by risk group in entire study population. **(A)** Disease-free survival, **(B)** Locoregional recurrence-free survival, **(C)** Distant metastasis-free survival, and **(D)** Disease-specific Overall Survival.

Of the 467 patients, 136 (29.31%) were in the low-risk group (T1 stage with one positive lymph node), 189 (40.73%) were in the intermediate-risk group (T1 stage with two to three positive lymph nodes, T2 stage with one positive lymph node, or T1 stage with ER-positive and HER2 positive tumor), and 139 (29.96%) were in the high-risk group (T2 stage with two to three positive lymph nodes or a tumor-proven immunohistochemically to be TNBC). There were significant differences in DFS (p = 0.036), DMFS (p = 0.0313), and OS (p = 0.006) among the three risk groups; LRRFS was not significantly different (p = 0.118) (**Figure 3**).

A total of 62 patients (45.59%) in the low-risk group, 88 patients (46.56%) in the intermediate-risk group, and 62 patients (44.60%) in the high-risk group were treated with WBI alone, whereas 74 patients (54.41%) in the low-risk group, 101 patients (53.44%) in the intermediate-risk group, and 77 patients (55.40%) in the high-risk group received WBI with RNI to axillary levels I–II. Receiving RNI significantly improved the 5-year LRRFS rate (86.82% vs. 97.22%, p = 0.014) in the high-risk group. There were no significant improvements in DFS, LRRFS, DMFS, or disease-specific OS in the low- and intermediate-risk groups.

### Case–control matched analysis in high-risk patients

With the development of radiation techniques, the number of patients receiving hypofractionated radiotherapy (23–25) using intensity-modulated radiation therapy with relatively higher fraction sizes has increased. Surgical advances have also led to changes in axillary lymph node surgery (26–28). We performed a case–control-matched analysis for each risk group to balance treatment characteristics between the two groups. Matching was performed based on the year of radiation exposure and the presence of TNBC. After case–control matched analysis, the low- and intermediate-risk groups did not show any differences in survival outcomes between the RNI group and those who received WBI alone. However, there were significant differences between the two groups in high-risk patients.

In the high-risk group, after case–control matched analysis, patient and treatment characteristics by radiation field were balanced between the two groups, without any statistically significant differences (**Table 3**). DFS and LRRFS between the RT with RNI group and the group with WBI alone were significantly different (p = 0.020 and p = 0.030, respectively); there was a marginal difference in DMFS (p = 0.066) and no difference in OS (p = 0.409) between the two groups (**Supplementary Figure S2**).

**Table 3:** Characteristics of patients and treatments in high-risk group after case–control matching.

	WBI, n = 24		RNI, n = 24		
	N, median	%, range	N, median	%, range	P-value
Age at RT					
Hypertension	3	12.50	5	20.83	0.439
Diabetes	2	8.33	1	4.17	0.551
Histology					0.429
IDC	21	87.50	20	83.33	
ILC	1	4.17	0		
others	2	8.33	4	16.67	
T stage					0.731
T1	6	25.00	5	20.83	
T2	18	75.00	19	79.17	
# of tumors					0.312
Single	24	100	23	95.83	
Multiple	0	0	1	4.17	
Lymphovascular invasion					0.551
Negative	8	33.33	10	41.67	
Positive	16	66.67	14	58.33	
Grade					0.226
1	1	4.17	3	12.50	
2	10	41.67	5	20.83	
3	13	54.17	16	66.67	
Margin status					0.303
Negative	17	70.83	20	83.33	
Close	7	29.17	4	16.67	
Lymph node dissection type					1.000
SLNB	5	20.83	5	20.83	
ALND	19	79.17	19	79.17	
Number of positive macroscopic nodes					0.470
1	10	41.67	8	33.33	
2	8	33.33	6	25.00	
3	6	25.00	10	41.67	
Hormone Receptor (+)	11	45.83	11	45.83	1.000
HER2 (+)	3	12.50	1	4.17	0.296
TNBC	13	54.17	13	54.17	1.000
RT technique					0.712
3D-CRT	19	79.17	20	83.33	
IMRT	5	20.83	4	16.67	
RT dose (cGy)					0.694
4,000–4,500	1	4.17	0		
5,005	5	20.83	5	20.83	
5,940	16	66.67	18	75.00	
6,480	2	8.33	1	4.17	
fraction size (cGy)					0.731
180	18	75.00	19	79.17	
267	6	25.00	5	20.83	
Adjuvant chemotherapy					1.000
No	2	8.33	2	8.33	
Yes	22	91.67	22	91.67	
Post RT chemotherapy					0.296
No	21	87.50	23	95.83	
Yes	3	12.50	1	4.17	
Anti-HER2 treatment					0.296
No	21	87.50	23	95.83	
Yes	3	12.50	1	4.17	

WBI, whole breast irradiation; RNI, regional nodal irradiation; RT, radiotherapy; IDC, invasive ductal carcinoma; ILC, invasive lobular carcinoma; SLNB, sentinel lymph node biopsy; ALND, axillary lymph node dissection; HER2, human epidermal growth factor receptor 2; TNBC, triple-negative breast cancer; 3D-CRT, 3D conformal radiotherapy; IMRT, intensity modulated radiotherapy.

### Toxicity

No grade 2 toxicities of pneumonitis or lymphedema were observed (**Table 4**). In the WBI alone group, 56 patients (26.42%) and one patient (0.47%) showed grade 2 and 3 skin dermatitis, respectively, whereas only 34 patients (13.49%) showed grade 2 skin dermatitis in the RNI with WBI group. After adjusting for the total radiation dose and fraction size, the incidence of skin dermatitis greater than grade 2 did not differ between the two groups (p = 0.995).

**Table 4:** Toxicity.

	Skin Dermatitis		Lymphedema		Radiation Pneumonitis	
	WBI	RNI	WBI	RNI	WBI	RNI
Grade 0	6 (2.83%)	9 (3.57%)	186 (87.74%)	238 (94.44%)	205 (96.70%)	251 (99.60%)
Grade 1	149 (70.28%)	209 (82.94%)	26 (12.26%)	14 (5.56%)	7 (3.30%)	1 (0.40%)
Grade 2	56 (26.42%)	34 (13.49%)	0	0	0	0
Grade 3	1 (0.47%)	0	0	0	0	0

WBI, whole breast irradiation; RNI, regional nodal irradiation.

## Discussion

This single-institution retrospective study suggests that omitting RNI should be cautiously considered in patients with pT1-2N1 breast cancer. Although the RNI did not show any significant differences in the entire cohort, it led to different survival outcomes when the patients were stratified into different risk groups. When RNI was defined as axillary lymph node levels I and II, it significantly improved DFS (p = 0.020) and LRRFS (p = 0.030), and marginally improved DMFS (p = 0.066) in the high-risk group. In this study, we attempted to define the extent of RNI as axillary lymph node levels I–II, and evaluated the use of RNI in different risk groups among pN1 patients who underwent BCS.

According to previous studies on patients with positive lymph node breast cancer (15, 29, 30), lymph node metastasis at axillary lymph node level I occurs in 20%–58% of patients, while only approximately 15%–31% of patients have metastasis at lymph node level III (30, 31). In addition, lymph node level IV, also defined as the supraclavicular lymph node (SCN), mostly drains through axillary lymph node levels I–III. Although there is direct nodal drainage to the SCN without passing through the axillary lymph node (32, 33), less than 10% of patients with pN1 breast cancer show lymph node level IV failure (34–37). Therefore, radiation administered for RNI in our institution included only axillary levels I and II for patients who did not have pathologically confirmed metastasis at lymph node level III/IV (6).

Survival outcomes in the WBI alone and RNI with WBI groups were comparable to those reported in previous studies. Trignani et al. (13) and Kim et al. (37) retrospectively evaluated survival outcomes in patients treated with WBI without SCN RT for breast cancer. In the study by Kim et al. (37), the 5-year DFS rates were 94.4% in the WBI alone group and 92.6% in the WBI with SCN RT group; the 5-year OS were 99.2% and 97.7%, the LRRFS were 98.1% and 96.1%, and the DMFS rates were 95.1% and 94.5%, respectively. Qi et al. (7) analyzed the survival outcomes of patients with pT1-2N1 breast cancer using data from two randomized controlled trials. In their study, the 5-year LRR rates were 2% and 5% in the WBI alone group vs. the WBI with RNI group, respectively, with 5-year DM rates of 7% and 13%. Sun et al. (19) also showed a 5-year LRR of 4.0% vs. 7.2%, DM of 13.2% vs. 10.6%, DFS of 85.0% vs. 84.7%, and OS of 93.9% vs. 92.8% in the RNI and non-RNI groups of patients with pT1-2N1 breast cancer, respectively. In these studies, all authors indicated that RNI was not necessary to improve the outcomes. However, a recent study (3) from the Early Breast Cancer Trialists’ Collaborative Group showed different results, estimating an absolute reduction of 2.7% in 15-year breast cancer mortality for patients with RNI in pN1 cases.

The criteria for identifying patients who would benefit the most from RNI and those who need to be cautiously selected for RNI omission are still under investigation. The results of the MA.20 study (1) and the EORTC 22922 trial (2) favored RNI, as the RNI group in both trials showed significantly improved 10-year DFS compared to the non-RNI group (86.3% vs. 82.4% and 72.1% vs. 69.1%, respectively). The MA.20 study also showed better loco-regional disease-free survival at 10 years in the RNI group, while the EORTC 22922 study showed better DMFS and breast cancer-related mortality at 10 years. However, with modern advances in surgical procedures, radiation technologies, and systemic treatment, the contribution of RNI to the reduction in recurrence might decrease in future prospective trials such as ongoing PORT-N1 and TAILOR RT studies (12). Furthermore, showing benefits not only in loco-regional control but also in DFS or DMFS might imply that applying RNI not only eradicates loco-regional tumor burden, but also blocks the dissemination of disease (19). To assess this, we stratified the patients into three risk groups based on their pathological T stage, number of positive lymph nodes, and immunohistochemical characteristics.

Few studies have evaluated the benefits of RNI in low-risk patients. The ongoing TAILOR RT by the Canadian Cancer Trials Group (NCT03488693) is expected to evaluate the benefit of RNI in biologically low-risk (ER-positive, HER2 negative, and Oncotype DX RS ≤25) breast cancer patients with one to three positive macroscopic nodes. Sit et al. (12) analyzed biologically low-risk breast cancer with criteria modeled from the TAILOR RT study using Oncotype DX for classifying the low-risk group. This retrospective study showed that RNI was not associated with an improvement in the breast cancer recurrence-free interval. Sun et al. (19) classified patients into three risk groups based on eight non-therapeutic risk factors: age, tumor location, pathologic T stage, number of positive nodes, LVI, histological grade, hormonal receptor, and HER2 status. These results are consistent with those reported by Sit et al. (12) In addition, our study did not show any statistical difference between the WBI alone and WBI with RNI groups in low-risk patients.

In contrast, our study demonstrated the beneficial effects of RNI in high-risk patients; in particular, when the two groups underwent case–control matched analysis, the difference became robust. After matching analysis, loco-regional recurrence-free survival (p = 0.030) and DFS (p = 0.020) were found to be statistically significant. Furthermore, the DMFS also showed a marginally significant difference (p = 0.066) between the WBI alone and WBI with RNI groups. Sun et al. (19) also mentioned that LRR had a beneficial effect on RNI in intermediate- and high-risk groups; however, in their study, the relative reduction in LRR from the RNI was greater in the intermediate-risk group, whereas our study did not show any survival differences in this risk group. In contrast to the insufficient use of systemic therapy reported by Sun et al., 97% of HER2-positive patients received anti-HER2 treatment and almost 75% of patients underwent adjuvant chemotherapy. Among patients who received adjuvant chemotherapy, 60% were treated with anthracycline plus taxane-based chemotherapy, resulting in better survival outcomes. It can be assumed that when there is sufficient systemic treatment, patients in high-risk groups may benefit more from RNI.

As this was a retrospective study, it had several limitations. First, despite our efforts to conduct a case–control matching analysis, the patients in these cohorts exhibited heterogeneity over different periods due to advancements in systemic treatment, surgery, and radiation techniques. This heterogeneity may have led to the application of different treatments based on changing guidelines, and ultimately, different patient outcomes. Second, the surgery type for the axillary lymph node was heterogeneous because there were more SLNB than ALND in the modern era. Although both SLNB and ALND were performed in the cohorts, this might not have a significant effect on survival outcomes, as several studies have shown that replacing ALND with SLNB is oncologically safe (38, 39). Third, a portion of levels I and II may have received radiation with the standard tangential field in the WBI alone. Given that this is a retrospective study aiming to bridge future prospective studies omitting RNI, the inclusion of the RNI field, with or without the intention of delivering at least 95% of the prescribed radiation dose, may be considered as a difference. Additionally, the use of the supine position for patient alignment could have introduced bias in the RNI group. Finally, there was a disproportion in the number of patients in the WBI alone and WBI with RNI groups. Although we attempted to overcome this limitation using a matching process, more balanced data between the two groups are needed in the future.

## Conclusion

In our study, RNI after BCS did not show any significant benefit on survival outcomes in low-risk groups. However, there was a robust benefit in terms of DFS, LRRFS, and DMFS in high-risk groups with T2 stage and two to three positive lymph nodes or tumors proven to be immunohistochemically TNBC. In addition, reducing the extent of RNI to axillary lymph node levels I–II did not lead to inferior survival outcomes and had comparable toxicities. While omitting RNI in low-risk patients can be considered, omitting RNI in high-risk patients needs to be cautiously examined, and reducing RNI to axillary lymph node levels I–II in patients with pN1 breast cancer who are at high risk after BCS might be considered. Future studies on the risk factors that benefit most from RNI as well as the extent of RNI should be conducted.

## Data Availability

The raw data supporting the conclusions of this article will be made available by the authors, without undue reservation.

## Supplementary material

Supplementary Figure 1 Flow chart. ALND, axillary lymph node dissection; RT, radiotherapy; SCN, supraclavicular lymph node; TNBC, triple-negative breast cancer.

Supplementary Figure 2 Survival Outcome in High Risk Group after Case-control Matching. **(A)** Disease-free survival **(B)** Locoregional recurrence-free survival **(C)** Distant metastasis-free survival **(D)** Disease-specific Overall Survival. WBI, whole breast irradiation; RNI, regional nodal irradiation.

## References

[1] Whelan TJ Olivotto IA Parulekar WR Ackerman I Chua BH Nabid A. Regional nodal irradiation in early-stage breast cancer. N Engl J Med. (2015) 373:307–16. doi: 10.1056/NEJMoa1415340 PMC4556358 26200977

[2] Poortmans PM Weltens C Fortpied C Kirkove C Peignaux-Casasnovas K Budach V. Internal mammary and medial supraclavicular lymph node chain irradiation in stage I-III breast cancer (EORTC 22922/10925): 15-year results of a randomised, phase 3 trial. Lancet Oncol. (2020) 21:1602–10. doi: 10.1016/S1470-2045(20)30472-1 33152277

[3] Early Breast Cancer Trialists' Collaborative Group (EBCTCG) Radiotherapy to regional nodes in early breast cancer: an individual patient data meta-analysis of 14 324 women in 16 trials. Lancet. (2023) 402:1991–2003. doi: 10.1016/S0140-6736(23)01082-6 37931633

[4] Sartor CI Peterson BL Woolf S Fitzgerald TJ Laurie F Turrisi AJ. Effect of addition of adjuvant paclitaxel on radiotherapy delivery and locoregional control of node-positive breast cancer: cancer and leukemia group B 9344. J Clin Oncol. (2005) 23:30–40. doi: 10.1200/JCO.2005.12.044 15545661

[5] Mannino M Yarnold JR. Local relapse rates are falling after breast conserving surgery and systemic therapy for early breast cancer: can radiotherapy ever be safely withheld? Radiother Oncol. (2009) 90:14–22. doi: 10.1016/j.radonc.2008.05.002 18502528

[6] Offersen BV Boersma LJ Kirkove C Hol S Aznar MC Biete Sola A. ESTRO consensus guideline on target volume delineation for elective radiation therapy of early stage breast cancer. Radiother Oncol. (2015) 114:3–10. doi: 10.1016/j.radonc.2014.11.030 25630428

[7] Qi WX Cao L Xu C Zhao S Chen J. Adjuvant regional nodal irradiation did not improve outcomes in T1-2N1 breast cancer after breast-conserving surgery: A propensity score matching analysis of BIG02/98 and BCIRG005 trials. Breast. (2020) 49:165–70. doi: 10.1016/j.breast.2019.11.001 PMC7375686 31812892

[8] Romond EH Perez EA Bryant J Suman VJ Geyer CE Jr. Davidson NE. Trastuzumab plus adjuvant chemotherapy for operable HER2-positive breast cancer. N Engl J Med. (2005) 353:1673–84. doi: 10.1056/NEJMoa052122 16236738

[9] Jang BS Shin KH. Postmastectomy radiation therapy in patients with minimally involved lymph nodes: A review of the current data and future directions. J Breast Cancer. (2022) 25:1–12. doi: 10.4048/jbc.2022.25.e6 35199499 PMC8876545

[10] Baranova A Krasnoselskyi M Starikov V Kartashov S Zhulkevych I Vlasenko V. Triple-negative breast cancer: current treatment strategies and factors of negative prognosis. J Med Life. (2022) 15:153–61. doi: 10.25122/jml-2021-0108 PMC8999097 35419095

[11] Lee TH Chang JH Jang BS Kim JS Kim TH Park W. Protocol for the postoperative radiotherapy in N1 breast cancer patients (PORT-N1) trial, a prospective multicenter, randomized, controlled, non-inferiority trial of patients receiving breast-conserving surgery or mastectomy. BMC Cancer. (2022) 22:1179. doi: 10.1186/s12885-022-10285-0 36384573 PMC9670382

[12] Sit D Lalani N Chan E Tran E Speers C Gondara L. Association between regional nodal irradiation and breast cancer recurrence-free interval for patients with low-risk, node-positive breast cancer. Int J Radiat Oncol Biol Phys. (2022) 112:861–9. doi: 10.1016/j.ijrobp.2021.10.149 34762971

[13] Trignani M Dic C Cefalogli C Nuzzo M Ursini LA Caravatta L. Outcomes in Patients with pT1-T2, pN0-N1 Breast Cancer After Conservative Surgery and Whole-breast Radiotherapy. In Vivo. (2017) 31:151–8. doi: 10.21873/invivo.11039 PMC5354142 28064235

[14] Mamounas EP Bryant J Lembersky B Fehrenbacher L Sedlacek SM Fisher B. Paclitaxel after doxorubicin plus cyclophosphamide as adjuvant chemotherapy for node-positive breast cancer: results from NSABP B-28. J Clin Oncol. (2005) 23:3686–96. doi: 10.1200/JCO.2005.10.517 15897552

[15] Veronesi U Rilke F Luini A Sacchini V Galimberti V Campa T. Distribution of axillary node metastases by level of invasion. An analysis of 539 cases. Cancer. (1987) 59:682–7. doi: 10.1002/1097-0142(19870215)59:4<682::AID-CNCR2820590403>3.0.CO;2-Z 3802027

[16] Rim CH Lee J Kim WC Yang D Yoon WS Koom WS. A survey of radiation therapy utilization in korea from 2010 to 2016: focusing on use of intensity-modulated radiation therapy. J Korean Med Sci. (2018) 33:e67. doi: 10.3346/jkms.2018.33.e67 29441739 PMC5811661

[17] Wang EH Mougalian SS Soulos PR Smith BD Haffty BG Gross CP. Adoption of intensity modulated radiation therapy for early-stage breast cancer from 2004 through 2011. Int J Radiat Oncol Biol Phys. (2015) 91:303–11. doi: 10.1016/j.ijrobp.2014.09.011 25442334

[18] De Santis MC Bonfantini F Dispinzieri M Meroni S Diletto B Mantero ED. Axillary coverage by whole breast irradiation in 1 to 2 positive sentinel lymph nodes in breast cancer patients. Tumori. (2016) 102:409–13. doi: 10.5301/tj.5000482 27002946

[19] Sun GY Wen G Zhang YJ Tang Y Jing H Fang H. Risk factors to identify the indication for regional nodal irradiation in T1-2N1M0 breast cancer: A joint analysis of 4,243 real-world cases from two institutions. Front Oncol. (2022) 12:955381. doi: 10.3389/fonc.2022.955381 36605447 PMC9807655

[20] Schlafstein A Liu Y Goyal S Kahn S Godette K Lin J. Regional nodal irradiation for clinically node-positive breast cancer patients with pathologic negative nodes after neoadjuvant chemotherapy. Clin Breast Cancer. (2022) 22:127–35. doi: 10.1016/j.clbc.2021.06.003 34229943

[21] Page DL. Prognosis and breast cancer. Recognition of lethal and favorable prognostic types. Am J Surg Pathol. (1991) 15:334–49. doi: 10.1097/00000478-199104000-00002 2006713

[22] Contesso G Jotti GS Bonadonna G. Tumor grade as a prognostic factor in primary breast cancer. Eur J Cancer Clin Oncol. (1989) 25:403–9. doi: 10.1016/0277-5379(89)90251-4 2649377

[23] Shaitelman SF Lei X Thompson A Schlembach P Bloom ES Arzu IY. Three-year outcomes with hypofractionated versus conventionally fractionated whole-breast irradiation: results of a randomized, noninferiority clinical trial. J Clin Oncol. (2018) 36:Jco1800317. doi: 10.1200/JCO.18.00317 30379626 PMC6286164

[24] Lertbutsayanukul C Pitak M Nantavithya C. Long-term oncological outcomes of hypofractionated versus conventional fractionated whole breast irradiation with simultaneous integrated boost in early-stage breast cancer. Radiat Oncol J. (2022) 40:141–50. doi: 10.3857/roj.2021.00927 PMC9262705 35796117

[25] Freedman GM White JR Arthur DW Allen Li X Vicini FA. Accelerated fractionation with a concurrent boost for early stage breast cancer. Radiother Oncol. (2013) 106:15–20. doi: 10.1016/j.radonc.2012.12.001 23333014

[26] Donker M van Tienhoven G Straver ME Meijnen P van de Velde CJ Mansel RE. Radiotherapy or surgery of the axilla after a positive sentinel node in breast cancer (EORTC 10981-22023 AMAROS): a randomised, multicentre, open-label, phase 3 non-inferiority trial. Lancet Oncol. (2014) 15:1303–10. doi: 10.1016/S1470-2045(14)70460-7 PMC4291166 25439688

[27] Veronesi U Paganelli G Viale G Luini A Zurrida S Galimberti V. A randomized comparison of sentinel-node biopsy with routine axillary dissection in breast cancer. N Engl J Med. (2003) 349:546–53. doi: 10.1056/NEJMoa012782 12904519

[28] Krag DN Anderson SJ Julian TB Brown AM Harlow SP Ashikaga T. Technical outcomes of sentinel-lymph-node resection and conventional axillary-lymph-node dissection in patients with clinically node-negative breast cancer: results from the NSABP B-32 randomised phase III trial. Lancet Oncol. (2007) 8:881–8. doi: 10.1016/S1470-2045(07)70278-4 17851130

[29] Rosen PP Lesser ML Kinne DW Beattie EJ. Discontinuous or “skip” metastases in breast carcinoma. Analysis of 1228 axillary dissections. Ann Surg. (1983) 197:276–83. doi: 10.1097/00000658-198303000-00006 PMC1352730 6830335

[30] Hu J Xia X Yang H Yu Y. Dissection of level III axillary lymph nodes in breast cancer. Cancer Manag Res. (2021) 13:2041–6. doi: 10.2147/CMAR.S290345 PMC7924124 33664591

[31] Yildirim E Berberoglu U. Lymph node ratio is more valuable than level III involvement for prediction of outcome in node-positive breast carcinoma patients. World J Surg. (2007) 31:276–89. doi: 10.1007/s00268-006-0487-5 17219275

[32] Tanis PJ Nieweg OE Valdés Olmos RA Kroon BB. Anatomy and physiology of lymphatic drainage of the breast from the perspective of sentinel node biopsy. J Am Coll Surg. (2001) 192:399–409. doi: 10.1016/S1072-7515(00)00776-6 11245383

[33] Estourgie SH Nieweg OE Olmos RA Rutgers EJ Kroon BB. Lymphatic drainage patterns from the breast. Ann Surg. (2004) 239:232–7. doi: 10.1097/01.sla.0000109156.26378.90 PMC1356216 14745331

[34] Yu JI Park W Huh SJ Choi DH Lim YH Ahn JS. Determining which patients require irradiation of the supraclavicular nodal area after surgery for N1 breast cancer. Int J Radiat Oncol Biol Phys. (2010) 78:1135–41. doi: 10.1016/j.ijrobp.2009.09.037 20231065

[35] Livi L Paiar F Simontacchi G Barca R Detti B Fondelli S. Loco regional failure pattern after lumpectomy and breast irradiation in 4,185 patients with T1 and T2 breast cancer. Implic nodal irradiation Acta Oncol. (2006) 45:564–70. doi: 10.1080/02841860600658211 16864170

[36] Truong PT Jones SO Kader HA Wai ES Speers CH Alexander AS. Patients with t1 to t2 breast cancer with one to three positive nodes have higher local and regional recurrence risks compared with node-negative patients after breast-conserving surgery and whole-breast radiotherapy. Int J Radiat Oncol Biol Phys. (2009) 73:357–64. doi: 10.1016/j.ijrobp.2008.04.034 18676091

[37] Kim H Park W Yu JI Choi DH Huh SJ Kim YJ. Prognostic impact of elective supraclavicular nodal irradiation for patients with N1 breast cancer after lumpectomy and anthracycline plus taxane-based chemotherapy (KROG 1418): A multicenter case-controlled study. Cancer Res Treat. (2017) 49:970–80. doi: 10.4143/crt.2016.382 PMC5654147 28052649

[38] Galimberti V Cole BF Viale G Veronesi P Vicini E Intra M. Axillary dissection versus no axillary dissection in patients with breast cancer and sentinel-node micrometastases (IBCSG 23-01): 10-year follow-up of a randomised, controlled phase 3 trial. Lancet Oncol. (2018) 19:1385–93. doi: 10.1016/S1470-2045(18)30380-2 30196031

[39] Giuliano AE Ballman KV McCall L Beitsch PD Brennan MB Kelemen PR. Effect of axillary dissection vs no axillary dissection on 10-year overall survival among women with invasive breast cancer and sentinel node metastasis: the ACOSOG Z0011 (Alliance) randomized clinical trial. Jama. (2017) 318:918–26. doi: 10.1001/jama.2017.11470 PMC5672806 28898379

