# Supplementary figures and images for: De-escalation of regional nodal irradiation fields in pT1-2N1 breast cancer patients after breast conserving surgery: retrospective real-world clinical experience
Source: Front Oncol. 2025 Mar 21;15:1484190. doi: 10.3389/fonc.2025.1484190 (PMC11968704; doi:10.3389/fonc.2025.1484190)

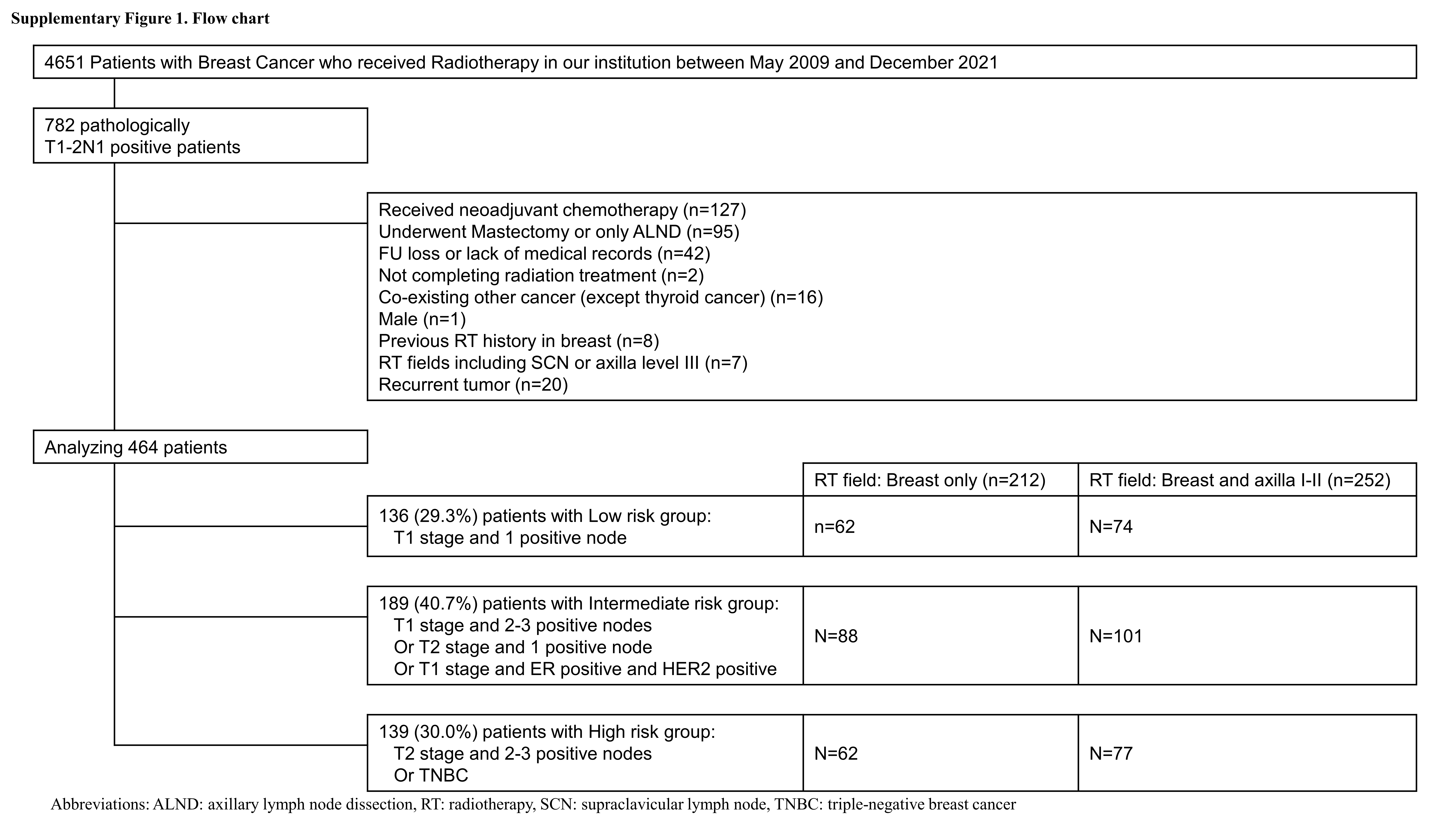

Supplement: Supplementary Figure 1 — Flow chart. ALND, axillary lymph node dissection; RT, radiotherapy; SCN, supraclavicular lymph node; TNBC, triple-negative breast cancer. [file Image1.tif]

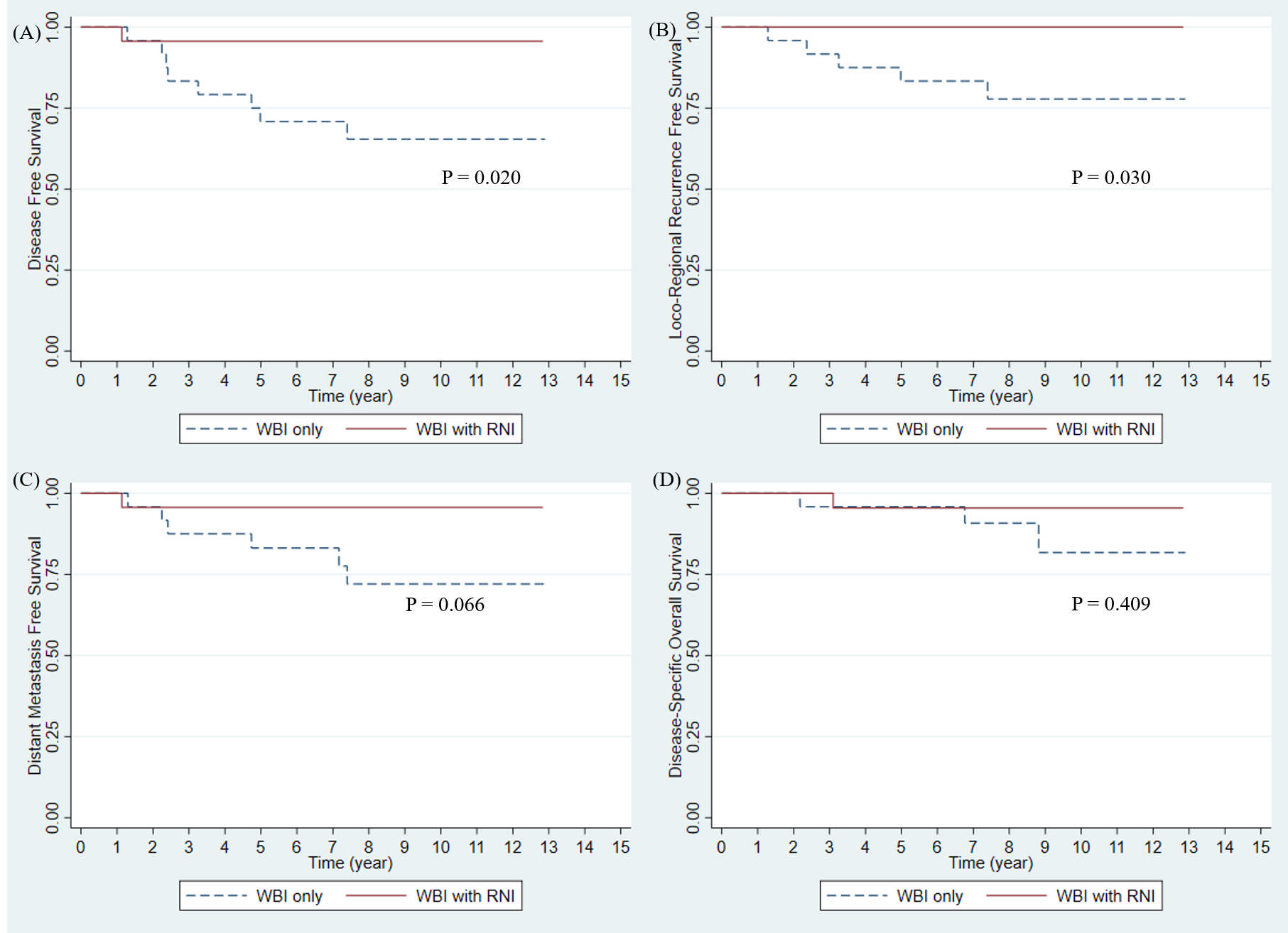

Supplement: Supplementary Figure 2 — Survival Outcome in High Risk Group after Case-control Matching. (A) Disease-free survival (B) Locoregional recurrence-free survival (C) Distant metastasis-free survival (D) Disease-specific Overall Survival. WBI, whole breast irradiation; RNI, regional nodal irradiation. [file Image2.tiff]
